# Association between food intake and mental health problems among preschoolers in China

**DOI:** 10.3389/fpubh.2022.1003416

**Published:** 2022-10-19

**Authors:** Qiyun Jin, Yi Liang, Junjie Miao, Yi Xiong, Zhijun Liu, Hongmei Xue, Zengning Li

**Affiliations:** ^1^Department of Child Healthcare, Zunyi Maternal and Child Health Care Hospital, Zunyi, China; ^2^Department of Clinical Nutrition, Affiliated Hospital of Guizhou Medical University, Guiyang, China; ^3^School of Public Health, Hebei Medical University, Shijiazhuang, China; ^4^Department of Applied Psychology, School of Management, Zunyi Medical University, Zunyi, China; ^5^Department of Clinical Nutrition, The First Hospital of Hebei Medical University, Shijiazhuang, China; ^6^Hebei Province Key Laboratory of Nutrition and Health, Shijiazhuang, China; ^7^College of Public Health, Hebei University, Baoding, China

**Keywords:** food category, food frequency, mental Health, SDQ, preschooler

## Abstract

**Objective:**

Our knowledge of associations between specific types of food and mental health problems in preschool children, an important developmental period, remains scarce. Using a large sample size and a brief measure, we aimed to examine associations between food groups and mental health problems in preschoolers.

**Methods:**

A descriptive cross-sectional study was conducted among 19,548 preschoolers in nine counties in China using a two-stage cluster sampling method. Information on food frequencies, physical activity, sedentary behavior, and sleep conditions was collected by a parent-reported questionnaire. Mental health status was assessed by a parent-reported Strength and Difficulty Questionnaire (SDQ). Height and weight were measured, and BMI was calculated. Relationships between specific types of food groups and mental health status were examined using multivariable regression modeling.

**Results:**

Of the present study sample, 47.45% of children were girls. Boys had higher scores relating to total difficulties, conduct problems, hyperactivity, and peer problems than girls but lower scores in emotional symptoms and prosocial behavior. Children with more types of food categories tended to get lower SDQ total scores even after adjusting for confounding variables (*p* < 0.0001). The subscale scores of SDQ varied depending on the types and numbers of food consumed. Notably, boys' mental health problems were more likely to be associated with their consumed food type. Processed meat frequency intake among boys and girls was positively related to prosocial behavior (Girls: pickled food: *p* = 0.003, fried food: *p* < 0.0001, barbecued food: *p* = 0.006, smoked food: *p* = 0.005; Boys: pickled food: *p* = 0.3, fried food: *p* < 0.0001, barbecued food: *p* = 0.006, smoked food: *p* = 0.005) and negatively associated with the SDQ total and the other subscale scores (Girls: pickled food: *p* ≤ 0.02, fried food: *p* ≤ 0.005 except for emotions (*p* = 0.1), barbecued food: *p* ≤ 0.047, smoked food: *p* ≤ 0.04 (except for emotions (*p* = 0.06) and conduct behaviors (*p* = 0.2)); Boys: pickled food: *p* < 0.01 except for hyperactivity (*p* = 0.5), fried food: *p* ≤ 0.005 except for emotions (*p* = 0.1), barbecued food: *p* ≤ 0.007 except for hyperactivity (*p* = 0.8), smoked food: p ≤ 0.001 except for hyperactivity (*p* = 0.1) and peer problem (*p* = 0.08)). Adequate intake frequency of confectionery (2–4 times/week) was beneficial to their emotions, conduct, and total difficulties and was also beneficial for peer and prosocial behavior for boys (Girls: emotions: *p* = 0.02, conduct: *p* = 0.02, hyperactivity: *p* = 0.4, peer: *p* = 0.06, total difficulties: *p* = 0.01, prosocial behavior: *p* = 0.1; Boys: emotions: *p* = 0.009, conduct: *p* = 0.0009, hyperactivity: *p* = 0.7, peer: *p* = 0.02: total difficulties: *p* = 0.003, prosocial behavior: *p* = 0.0002).

**Conclusion:**

Diverse food categories and adequate frequency of confectionery were found to be beneficial for children's mental health, with the exception of processed meat. These findings provide novel information and supportive data for children's dietary guidelines and the improvement of mental health.

## Introduction

Mental health disorders that affect children are of serious concern (1, 2) due to their persistent impacts on various areas of life, including personal relationships and physical health (3, 4). Globally, it is estimated that 10–20% of children and adolescents experience a mental disorder in their lifetime (2, 5). In China, approximately 70% of children have at least one mental health problem (6). Multiple factors affect a child's mental health, and these may include ethnic or sexual backgrounds (2), parental disorders and parenting style (7–9), media influence (10–13), physical activity (11, 13, 14), sleep disorders (15, 16), and dietary behaviors (17, 18).

An individual's diet is also well suggested to be a contributing factor that may affect mental health. A meta-analysis conducted by Adrienne O'Neil et al. in 2014 found that the frequency of eating breakfast and fruit intake demonstrated a cross-sectional relationship between unhealthy dietary patterns and poor mental health in children and adolescents (17). Khalid et al. reviewed 12 studies in 2016, in which their overall findings supported an association between healthy dietary patterns or consumption of a high-quality diet and better mental health. Głabska et al. (19) conducted a meta-analysis in 2019, suggesting that vitamin D intake within a properly balanced diet or as supplementation was indicated as an essential element supporting children's mental health. Additionally, fatty fish intake has been observed to support the traditional pharmacologic interventions for mental health problems, verified by randomized controlled trials in children (20). Furthermore, a systematic review conducted in 2020 suggested a positive influence of fruit and vegetable intake on children's mental health (21). Thus, it is well supported that improving diet may help protect the individual's physical health and the population's mental health (22). Based on the available evidence above, we could conclude that a balanced diet maintains better wellbeing and lowers the risk of mental disorders in children and adolescents. However, a clear definition of a balanced diet for preschoolers, a critical period for social character formation and emotional instability, remains to be elucidated.

Studies of diet and mental health problem among Chinese children are emerging but sparse. One study focused on preschoolers in rural China suggested that dietary diversity was related to improving children's mental health (6). Liang et al. centered on left-behind children and found that lower frequencies of eating breakfast and fruits were significant factors associated with higher risks of mental health problems (23). Epidemiological evidence based on large populations and centered around specific food categories is desiderated.

With regards to these considerations, the present study aimed to investigate the relationship of specific food categories like cereals/tubers, vegetables and fruits, dairy/soy/nuts, meat/poultry/fish/eggs, and processed meats with mental health among 19,548 preschoolers from Zunyi, China, a gathering place of multi-ethnic residents.

## Materials and methods

### Study population

We collected data from August and September 2020 as part of the baseline survey of an ongoing cohort study. Preschoolers aged 3.0–7.0 years old were from 9 counties (3 municipal districts: Honghuagang District, Huichuan District, Bozhou District; 5 counties: Meitan County, Tongzi County, Zunyi County, Xishui County, Shuiyang County; and 1 minority autonomous county: Daozhen County) of Zunyi City, Guizhou Province, China. These preschoolers were recruited using a stratified random sampling method with the distributions of age, gender, and annual family income similar to children in the general population of urban and rural areas (24). For this study, we draw on five types of information collected by the survey team: (a) Basic information about children and families from caregivers, including socioeconomic data, conditions of birth and delivery, and disease history; (b) activities and dietary intake, including food frequencies, physical activity, and sedentary behavior; (c) nutritional status measurement, including height and weight; (d) scores on sleep problem as assessed by the Children's Sleep Habits Questionnaire (CSHQ), and (e) scores on mental health as assessed by the Strengths and Difficulties Questionnaire (SDQ). The study followed the principles of the Declaration of Helsinki and was approved by the Ethics Committee of Zunyi Maternal and Child Health Care Hospital (No. 202110). All examinations were performed with the written consent of parents or guardians.

Initially, 19,660 children were recruited. Of those recruited, 112 children with incomplete data (missing anthropometric data or information on potential confounders) were excluded. Therefore, this analysis is based on a final sample of 19,548 participants (9,275 females, 47.45%; 10,273 males, 52.55%).

### Measurement of mental health problem

Children's mental health status was assessed through the SDQ (25). As a valid and reliable behavioral screening questionnaire (10, 26), the SDQ consists of 25 items and includes 5 subscales: emotional symptoms, hyperactivity, conduct problems, peer problems, and prosocial behavior. Each subscale includes five questions with three possible responses: not true (assigned value of 0), somewhat true (assigned value of 1), or certainly true (assigned value of 2). Scores for each subscale are summed to a total ranging from 0 to 10. Higher scores indicate more problems, except for the prosocial behavior subscale, for which a lower score indicates more problems. The total difficulties score (ranging from 0 to 40) is calculated by the total sum of the scores on the first four aforementioned subscales to assess children's behavioral problems. The SDQ is validated in cross-cultural settings and for diagnoses of mental health problems among children in clinical and research settings in China and abroad.

In the present study, an electronic questionnaire of the SDQ was sent to both parents and teachers. Firstly, the parent (usually the mother) was asked to complete the SDQ and submit it within a specific time limit. Following the parental questionnaire, the pediatrician sent the questionnaire to the teacher for completion. Of all children who participated in the present study, the SDQ was completed by 85.58% of teachers (*n* = 16,729).

### Dietary intake

In our study, total and specific food groups consumed per day or week were administered to the parents. Based on dietary guidelines for Chinese residents, we classified food groups as cereals and tubers, vegetables and fruits, meat/poultry/fish/eggs, dairy/soy/nuts, and vegetable oil. To estimate how often foods were consumed on average by their child over the previous year, parents were asked to choose one of three frequency categories, ranging from “one type per week” to “ten types or more per week.” Total number of food groups consumed per week was also asked by setting five options ranging from “six or fewer types per week” to “twenty-five or more types per week.” Furthermore, a processed food category (pickled food, fried food, barbecued food, smoked food, and confectionery) was also designed to measure the children's usual food intake over the past year. The corresponding dietary questionnaire were displayed in the Supplementary Table S1.

### Covariates

We assessed confounding factors potentially associated with diet and mental health status for the present analysis. These included basic information on children (gender, birth date, birth weight, and gestational age), socio-demographic data (parental education levels, parental employment, household income), childhood overweight/obesity, physical activity, screen time, and sleep problems assessed by the CSHQ (27).

Health care practitioners from the kindergartens measured the height and weight of pre-schoolers. BMI was calculated, and the definition of overweight childhood and obesity were defined according to the BMI reference standard of Chinese children and adolescents aged 2–18 years old as proposed by Li et al. (28). This proposed definition classifies children as overweight and/or obese when the BMI is greater than or equal to the overweight cut-off point for the same sex and age (in line with adults).

Considering the close relationship between sleep problems and mental health (29), we assigned the CSHQ scale, which had been validated among Chinese children (30). To assess the sleep conditions and information on children's time spent on outdoor activities and screens (TV, PC, and DVD) at home, their parents provided the average time spent on these activities per day.

### Statistical analysis

SAS procedures (SAS, version 9.4, 2012, SAS Institute Inc., Cary, NC, USA) were used for data analyses. All analyses were performed with a significance level of *p* < 0.05, except for interaction tests, where *p* < 0.1 was considered significant. All continuous variables' normality was examined using normal probability plots and the Kolmogorov-Smirnov test. In consideration of differential development of body composition throughout growth in boys and girls, the subsequent analyses in this study were performed for girls and boys separately.

Differences in SDQ scores, food groups, and confounders between boys and girls were tested using ANOVA for normally distributed continuous variables and chi-square tests for categorical variables.

Multivariate linear regression models were used to investigate the association of food groups with the SDQ scores. In the present analysis, the SDQ scores of difficulties and emotional symptoms, hyperactivity, conduct disorders, peer problems, and prosocial behavior scores were considered dependent variables. Total food types, as well as specific food categories including cereals and tubers, vegetables and fruits, meat/poultry/fish/eggs/, dairy/soy/nuts, portion of vegetable oil, pickled food, fried food, barbecued food, smoked food, and confectionery, were defined as the independent variable in separate models.

The correlation analyses between each food category and the SDQ scores were first carried out in the basic models. In a later step, potential covariates that may affect these associations were added, which included: continuous variables—age (years), birth weight (kg), gestational age (weeks), and CSHQ score; and categorical variables—nation (Han, other nations), residence (rural, town, city), number of children (one child, 2-3 children, 4-5 children), parental education level (<6 years, 6–12 years, and >12 years of schooling) maternal employment (manual, mental worker), average annual household income (<6,000 CNY, 6,000–40,000 CNY, ≥40,000 CNY), time spent on outdoor activities (<2 h/day, 2–4 h/day and ≥4 h/day), screen time (<1 h/day, 1–2 h/day and ≥2 h/day), and BMI categories (normal, overweight and obesity). Only variables that had a significant independent effect in the basic models or those that substantially altered the association between the food categories and the SDQ scores were involved in the subsequent multivariable analyses. The adjusted means were the least-squares means predicted by the model when the other variables were held at their mean values.

## Results

### General characteristics

Of the present study sample, 47.45% of children were girls, 76.99% were of Han nationality, and the mean age was 4.59 ± 0.93 years (Table 1). The prevalence of overweight and obesity among boys was higher than that of girls (overweight: 12.88 vs. 8.54%; obesity: 13.74 vs. 8.93%, all *p* < 0.0001). Boys spent an average of ≥2 h/day on screen and ≥2 h/day on outdoor activities, demonstrating higher averages spent on these activities than girls (*p* < 0.005). As for SDQ scores, scores of total difficulties, conduct problems, hyperactivity, and peer problems were higher in boys than girls. Likewise, they had lower scores in emotional symptoms and prosocial behavior than that for girls (*p* < 0.02).

**Table 1:** Characteristics of study sample by sex^a^ (*n* =19,548).

**Group**		**Female**	**Male**	***p***
**Child characteristics**				
*n* (%)		9,275 (47.45)	10,273 (52.55)	-
Nation (Han, %)		7,089 (76.43)	7,960 (77.48)	0.3
Age (yrs)		4.56 ± 0.91	4.61 ± 0.94	0.003
Gestational age (weeks)		38.29 ± 3.21	38.23 ± 3.15	< 0.0001
Siblings (%)	One child	2,149 (10.99)	2,677 (13.69)	< 0.0001
	2–3 children	7,032 (35.97)	7,515 (38.44)	
	4–5 children	94 (0.48)	81 (0.41)	
Residence (%)	Rural	3,182 (16.28)	3,508 (17.95)	0.2
	Town	1,712 (8.76)	1,806 (9.24)	
	City	4,381 (22.41)	4,959 (25.37)	
Birthweight (kg)		3.14 ± 0.55	3.26 ± 0.57	< 0.0001
Weight (kg)		16.00 ± 3.42	16.84 ± 3.70	< 0.0001
Height (cm)		104.55 ± 10.09	105.62 ± 10.38	< 0.0001
Body mass index (kg/m^2^)		14.79 ± 3.44	15.26 ± 3.66	< 0.0001
BMI categories (%)^b^	Normal weight	7,785 (39.83)	7,859 (40.20)	< 0.0001
	Overweight	727 (3.72)	1,162 (5.94)	
	Obesity	763 (3.90)	1,252 (6.40)	
Screen time (%)	< 1 h/day	4,282 (21.91)	4,442 (22.72)	< 0.0001
	1–2 h/day	3,539 (18.10)	3,904 (19.97)	
	≥2 h/day	1,454 (7.44)	1,927 (9.86)	
Outdoor activity (%)	< 2 h/day	6,033 (30.86)	6,467 (33.08)	0.005
	2–4 h/day	2,701 (13.82)	3,133 (16.03)	
	≥4 h/day	541 (2.77)	673 (3.44)	
CSHQ score		51.49 ± 5.89	51.51 ± 5.85	0.7
SDQ score^c^	Total difficulties	10.02 ± 3.84	10.43 ± 3.73	< 0.0001
	Emotional symptoms	2.20 ± 1.85	2.13 ± 1.78	0.02
	Conduct problems	1.24 ± 0.97	1.33 ± 0.99	< 0.0001
	Hyperactivity	4.06 ± 1.71	4.39 ± 1.72	< 0.0001
	Peer problems	2.51 ± 1.12	2.58 ± 1.11	< 0.0001
	Prosocial behavior	6.83 ± 1.96	6.43 ± 2.01	< 0.0001
**Parental characteristics**				
Parental High education level^d^		5,014 (54.06)	5,610 (54.61)	0.4
Paternal employment (Manual, %)		4,082 (44.01)	4,506 (43.48)	0.8
Maternal employment (Manual, %)		2,895 (31.21)	3,405 (33.15)	0.004
Average annually household income (%)^e^			
Low		2,903 (14.85)	3,095 (15.83)	0.002
Medium		3,419 (17.49)	3,661 (18.73)	
High		2,953 (15.11)	3,517 (17.99)	
COVID (non-impact, %)				

^a^Values Are Mean ± SD. P Values Are Calculated From Chi-Square Test for Categorical Variables and Analysis of Variance for Continuous Variables.

^b^Calculated According to China Criteria Developed by Capital Institute of Pediatrics (29).

^c^Calculated Based on Strengths and Difficulties Questionnaire (28).

^d^School Year ≥12.

^e^Low Income: < 6,000 CNY; Medium: 6,000–40,000 CNY; High: ≥40,000 CNY.

### Association between food varieties and SDQ scores among boys

Among boys, children with an intake of more food categories tended to get lower SDQ total scores, which suggests better mental health. Compared to children with ≤ 6 types/week of food categories, those eating more than 12 different foods per week had a statistically significant lower SDQ total score (6.81%; all *p* < 0.0001). The same trends were observed with the intake of cereals and tubers, vegetables and fruits, meat/poultry/fish/eggs, and dairy/soy/nuts (Table 2).

**Table 2:** Multiple linear regression analysis between food varieties and SDQ total score^a^ (*n* = 19,548).

**Food category**	**Female**				**Male**			
	**Mean (SE) of SDQ total score**				**Mean (SE) of SDQ Total score**			
	**Unadjusted**	**Model 1^b^**	**Model 2^c^**	**Model 3^d^**	**Unadjusted**	**Model 1^b^**	**Model 2^c^**	**Model 3^d^**
**All foods (types/week)**							
≤ 6	10.49 (0.06)	10.65 (0.22)	11.14 (0.26)	11.11 (0.24)	10.82 (0.06)	10.62 (0.22)	10.87 (0.24)	11.01 (0.23)
7–11	9.85 (0.06)	10.16 (0.22)	10.62 (0.25)	10.59 (0.24)	10.24 (0.06)	10.18 (0.22)	10.39 (0.24)	10.57 (0.23)
≥12	9.50 (0.09)	9.77 (0.24)	10.25 (0.26)	10.26 (0.25)	10.03 (0.08)	9.87 (0.24)	10.10 (0.25)	10.26 (0.24)
*p* value	< 0.0001	< 0.0001	< 0.0001	< 0.0001	< 0.0001	< 0.0001	< 0.0001	< 0.0001
**Cereals/tubers (types/week)**						
1–2	10.22 (0.06)	10.39 (0.22)	10.80 (0.25)	10.79 (0.24)	10.68 (0.06)	10.58 (0.22)	10.78 (0.24)	10.88 (0.23)
3–4	9.93 (0.06)	10.12 (0.22)	10.56 (0.25)	10.50 (0.23)	10.31 (0.06)	10.18 (0.22)	10.42 (0.24)	10.60 (0.23)
≥5	9.74 (0.10)	10.15 (0.25)	10.65 (0.28)	10.71 (0.26)	10.05 (0.10)	9.85 (0.25)	10.12 (0.26)	10.35 (0.25)
*p* value	< 0.0001	0.2	0.3	0.09	< 0.0001	< 0.0001	0.0004	0.006
**Vegetables/fruits (types/week)**						
1–3	10.39 (0.07)	10.65 (0.23)	11.11 (0.26)	11.04 (0.24)	10.78 (0.06)	10.61 (0.22)	10.84 (0.25)	10.91 (0.23)
4–6	10.09 (0.06)	10.35 (0.21)	10.82 (0.25)	10.76 (0.24)	10.34 (0.06)	10.20 (0.22)	10.43 (0.24)	10.64 (0.22)
≥7	9.39 (0.08)	9.53 (0.23)	10.04 (0.26)	10.13 (0.25)	10.11 (0.08)	10.00 (0.24)	10.24 (0.25)	10.41 (0.24)
*p* value	< 0.0001	< 0.0001	< 0.0001	< 0.0001	< 0.0001	0.0005	0.0007	0.006
**Meat/poultry/fish/eggs (types/week)**						
≤ 1	10.83 (0.09)	10.90 (0.25)	11.32 (0.28)	10.36 (0.26)	11.05 (0.08)	10.82 (0.24)	11.08 (0.26)	11.21 (0.25)
2–4	9.93 (0.05)	10.19 (0.20)	10.64 (0.24)	10.59 (0.23)	10.34 (0.04)	10.21 (0.21)	10.42 (0.23)	10.59 (0.22)
≥5	9.32 (0.12)	9.64 (0.27)	10.13 (0.30)	10.21 (0.28)	9.94 (0.11)	9.84 (0.27)	10.13 (0.28)	10.29 (0.27)
*p* value	< 0.0001	< 0.0001	< 0.0001	< 0.0001	< 0.0001	< 0.0001	< 0.0001	< 0.0001
**Dairy/soy/nuts (types/week)**						
≤ 1	10.66 (0.08)	10.78 (0.24)	11.20 (0.27)	11.14 (0.25)	10.87 (0.07)	10.74 (0.23)	10.95 (0.25)	11.05 (0.24)
2–4	9.88 (0.05)	10.13 (0.21)	10.57 (0.24)	10.55 (0.23)	10.31 (0.05)	10.12 (0.21)	10.34 (0.23)	10.52 (0.22)
≥5	9.56 (0.12)	9.79 (0.28)	10.26 (0.30)	10.32 (0.29)	10.15 (0.11)	10.18 (0.27)	10.43 (0.29)	10.57 (0.27)
*p* value	< 0.0001	< 0.0001	< 0.0001	0.0001	< 0.0001	< 0.0001	0.0001	0.0005
**Vegetable oils proportion**							
≥2/3	10.00 (0.05)	10.19 (0.21)	10.63 (0.25)	10.66 (0.23)	10.39 (0.05)	10.25 (0.22)	10.47 (0.23)	10.67 (0.22)
1/3–2/3	9.91 (0.07)	10.21 (0.22)	10.64 (0.25)	10.56 (0.24)	10.27 (0.06)	10.04 (0.24)	10.24 (0.24)	10.36 (0.23)
< 1/3	10.43 (0.12)	10.45 (0.27)	10.90 (0.30)	10.85 (0.28)	11.07 (0.11)	10.96 (0.26)	11.15 (0.27)	11.26 (0.26)
*p* value	0.0007	0.5	0.4	0.4	< 0.0001	< 0.0001	< 0.0001	< 0.0001

^a^Values Presented in the Table Are Least-Squares Means.

^b^Model 1 Adjusted for age, Gestation age, Nation, Resident, Siblings' Number, Maternal Employment, Average Annually Household Income, Birthweight.

^c^Model 2: Model 1 Additionally Adjusted for BMI Categories, Outdoor Activity, Screen Time.

^d^Model 3: Model 2 Additionally Adjusted for CSHQ Score.

**Figure 1:**
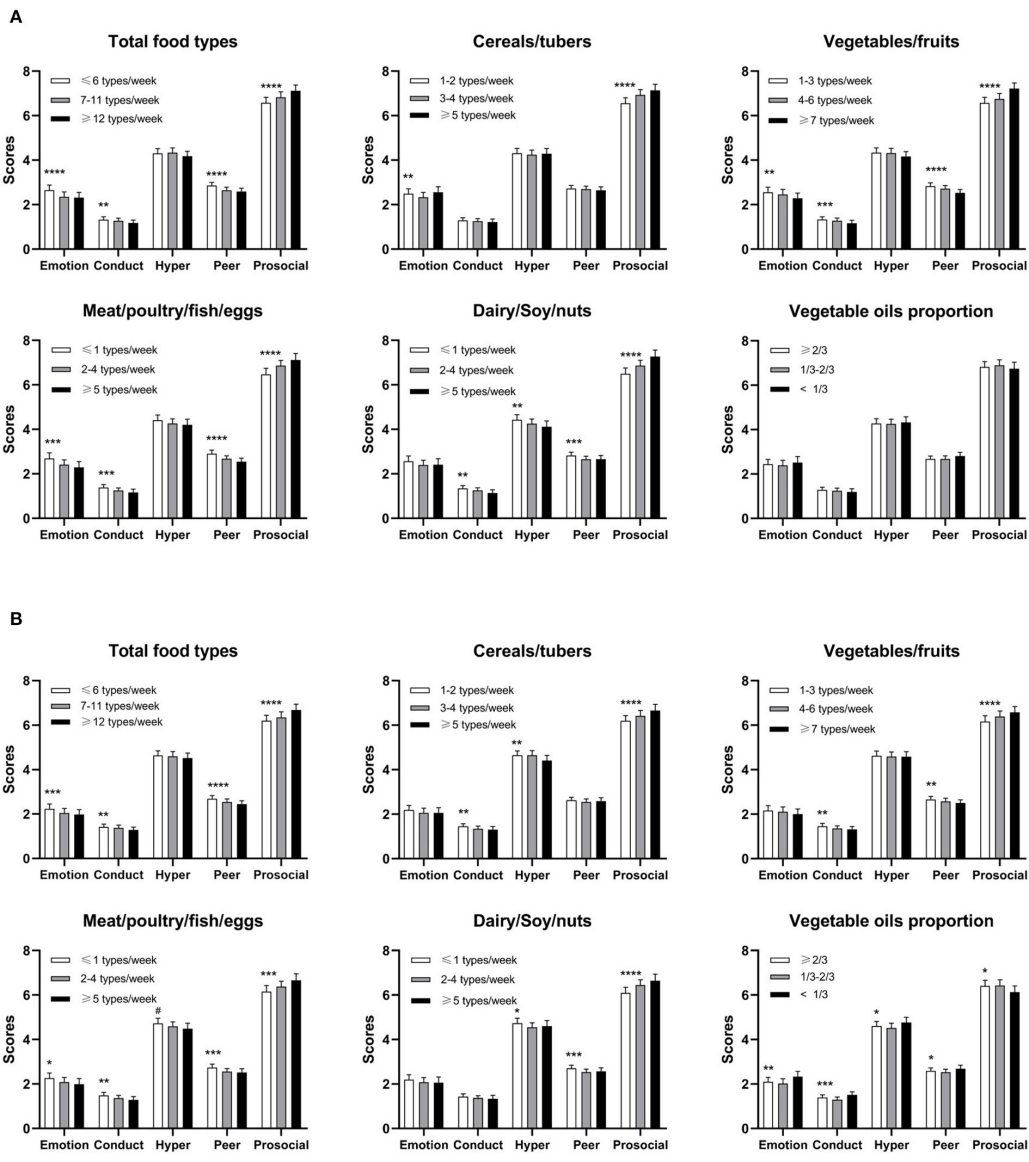
Multiple linear regression analysis between food varieties and the SDQ subscale scores (*n* = 19,548); **(A)** for girls and **(B)** for boys. Data shown in the figure were adjusted for age, gestation age, nation, resident, siblings' number, maternal employment, average annually household income, birthweight, BMI categories, outdoor activity, screen time, and CSHQ; ****p < 0.0001, ***p < 0.001, **p < 0.01, *p < 0.05, and ^#^*p* = 0.051.

The relationship between food varieties and the SDQ subscale scores was also analyzed (Figure 1), and we found that the subscale scores of SDQ varied depending on the types and numbers of food consumed. Boys who consumed more cereals/tubers were reported to have less conduct, hyperactivity, and prosocial problems. Those that consumed more vegetables/fruits were found to have less conduct and peer and prosocial problems. Additionally, those consuming more meat/poultry/fish/eggs had less emotional symptoms, conduct, and peer and prosocial problems. Finally, those who consumed more dairy/soy/nuts had less conduct, hyperactivity, and peer and prosocial problems.

In the subsequent analysis, we analyzed the effect of processed meat on SDQ total and subscale scores. Pickled, fried, barbecued, and smoked food intake frequencies were negatively associated with the SDQ total and subscale scores (pickled food: *p* < 0.01 except for hyperactivity (*p* = 0.5), fried food: *p* ≤ 0.005 except for emotions (*p* = 0.1), barbecued food: *p* ≤ 0.007 except for hyperactivity (*p* = 0.8), smoked food: *p* ≤ 0.001 except for hyperactivity (*p* = 0.1) and peer problem (*p* = 0.08)), except for prosocial behavior, which was positively associated (pickled food: *p* = 0.3, fried food: *p* < 0.0001, barbecued food: *p* = 0.006, smoked food: *p* = 0.005). Interestingly, we found a U-shaped association between confectionery intake frequency and emotional symptoms, conduct problems, peer problems, and total difficulties. In contrast, we found an invertedly U-shaped association for prosocial behavior. These findings suggest that too much or little confectionery intake frequencies were detrimental for children's mental health (emotions: *p* = 0.009, conduct: *p* = 0.0009, hyperactivity: *p* = 0.7, peer: *p* = 0.02: total difficulties: *p* = 0.003, prosocial behavior: *p* = 0.0002).

### Association between food varieties and SDQ scores among girls

Similar to boys, wider food varieties were negatively associated with the SDQ total scores after adjusting for confounding variables. That is to say, a wide variety of food intake was good for girls' mental health (*p* < 0.0001). In addition, girls with more kinds of vegetables/fruits, meat/poultry/fish/eggs, and dairy/soy/nuts also had fewer total difficulties. We also found that girls with more kinds of cereals/tuber had fewer emotional problems and were prosocial. Girls who consumed more kinds of vegetables/fruits and meat/poultry/fish/eggs had less emotional problems, conduct, peer and prosocial problems. Finally, those who consumed more dairy/soy/nuts had less conduct, hyperactivity, peer and prosocial problems (*p* < 0.01). No associations were observed between vegetable oil proportion and the SDQ subscale score.

**Figure 2:**
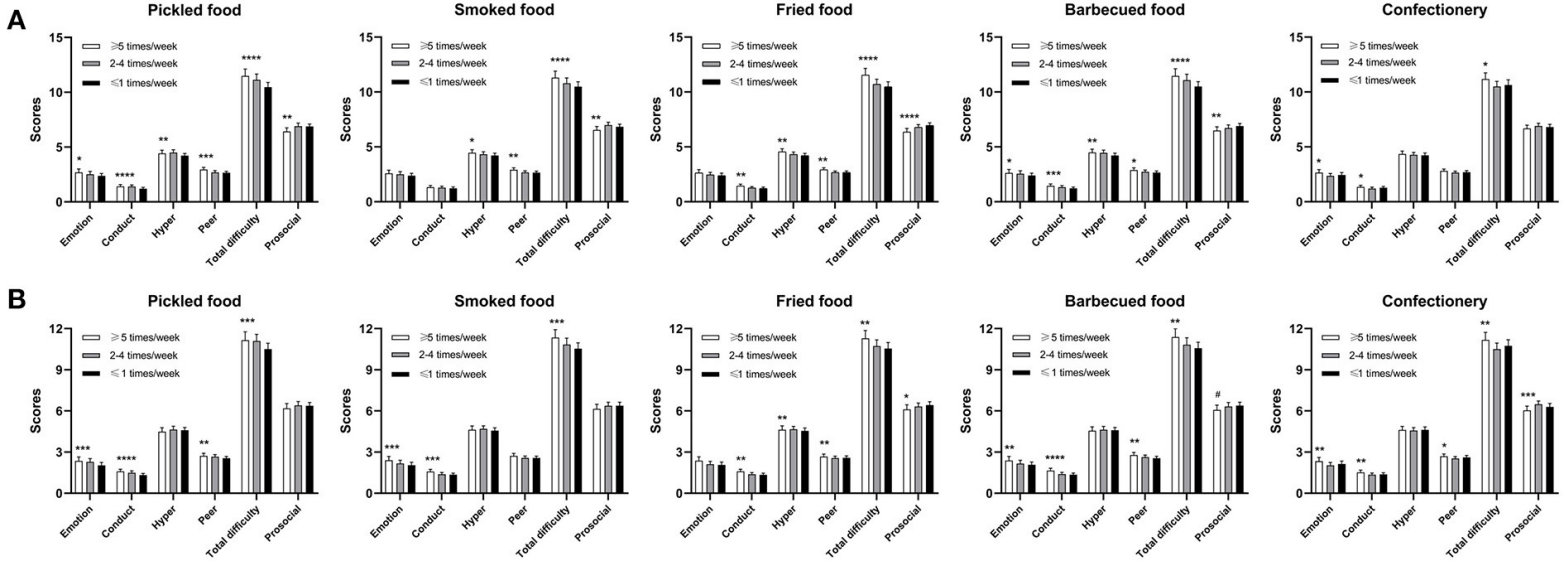
Multiple linear regression analysis between processed food and the SDQ subscale scores (*n* = 19,548); **(A)** for girls and **(B)** for boys. Data shown in the figure were adjusted for age, gestation age, nation, resident, siblings' number, maternal employment, average annually household income, birthweight, BMI categories, outdoor activity, screen time, and CSHQ; ****p < 0.0001, ***p < 0.001, **p < 0.01, *p < 0.05, and ^#^*p* = 0.054.

Pickled and barbecued food frequencies were positively related to the prosocial behavior scores (pickled food: *p* = 0.003, fried food: *p* < 0.0001, barbecued food: *p* = 0.006, smoked food: *p* = 0.005) and negatively related to the SDQ total and other subscale scores (pickled food: *p* ≤ 0.02, fried food: *p* ≤ 0.005 except for emotions (*p* = 0.1), barbecued food: *p* ≤ 0.047, smoked food: *p* ≤ 0.04 except for emotions (*p* = 0.06) and conduct behaviors (*p* = 0.2)) (Figure 2). Girls with higher fried food and smoked food intake frequency were found to have less hyperactivity, peer and prosocial behavior, and total difficulties (*p* < 0.05). Additionally, fried food frequency intake was also negatively associated with conduct problems (*p* = 0.003). Similar to boys, we found girls with adequate intake frequency of confectionery (2–4 times/week) is beneficial to girls' emotion, conduct, and total difficulties (emotions: *p* = 0.02, conduct: *p* = 0.02, hyperactivity: *p* = 0.4, peer: *p* = 0.06, total difficulties: *p* = 0.01, prosocial behavior: *p* = 0.1).

## Discussion

The present study found that food varieties were associated with better mental health in boys and girls of preschool age. Preschoolers that consumed more cereals/tubers, vegetables/fruits, meat/poultry/fish/eggs, and dairy/soy/nuts had fewer mental health problems, especially prosocial behavior, peer problems, and conduct problems. In contrast, high-frequency consumption of processed meat was detrimental to children's mental health conditions, particularly in the observed total difficulties, boys' conduct problems, and girls' peer problems. Simultaneously, adequate frequency of confectionery may be beneficial for children's mental health. Our findings provide novel information and supportive data for the scheme of children's dietary guidelines and improving their mental health.

As mentioned above, a properly balanced diet, the number and proportion of different types of food in the diet, and the frequency of consumption allow for maintaining better wellbeing and lowers the risk of mental disorders in children and adolescents (21, 31, 32). It is recommended by the Dietary Guidelines for Chinese Residents in 2016 that preschool children should eat an average of more than 12 kinds of food per day and 25 kinds of food per week (33). In the present study, only 4.62% of preschoolers met these recommendations (data not shown), in which they were reported to eat an average of more than 25 foods per week. We also found that eating more than 12 types of food was significantly beneficial for boys' and girls' psychosocial development, which is consistent with the studies on rural Chinese preschoolers (6), which focused on dietary diversity and suggested that this diversity was an important strategy to improve child mental health. Upon further analysis, we found that the association between food categories and aspects of mental health were similarly significant. Therefore, ensuring the intake of food types could be an important strategy to consider in designing interventions to improve child mental health.

The Mediterranean Diet (MD) is currently considered one of the healthiest dietary models worldwide, generally based on the daily intake of fruit and vegetables, whole grains, legumes, nuts, fish, white meats, and olive oil (34). Emerging evidence on the possible impact of MD on mental health has been examined and reported by some studies (35). Consistent with this finding, we revealed the benefits of the specific types of food composition of MD.

Vegetables and fruits are an essential part of the MD. Numerous studies (21, 36, 37) indicate that vegetables and/or fruits have a positive influence on mental health. Consistent with previous studies, our findings showed that preschoolers with an intake of vegetables/fruit for more than or equal to 7 types per week may have improved mental health. The mechanism of vegetables and fruit influence on mental health is still unknown. However, these effects may be dependent on anti-inflammatory mechanisms and the specific nutrients they contain, like complex carbohydrates (38), fiber (39), vitamin B (40), and vitamin C (41) being associated with a stable glycemic index, and the presence of carotenoids (42) and polyphenols (43).

Cereal and tuber consumption, providing a large amount of dietary fiber and a good source of vitamins B and carbohydrates, are parts of an overall healthful lifestyle. However, whether cereals and tubers are associated with mental health is needed to be further studied. Some studies have demonstrated that cereal crops can act as a protective factor beneficial for the mental health of adults (44). In contrast, some studies found null relations between cereals/tubers consumption and mental health among older adults (45) and students (36). Regardless, no studies were conducted among preschoolers. This study suggested that cereal/tubers were negatively associated with conduct problems, hyperactivity, prosocial behavior, and total difficulties in boys. Likewise, our findings suggest that they are negatively associated with emotion syndrome and prosocial behavior of girls. Therefore, keeping the intake of cereals/tubers ≥5 types per week may benefit mental health development.

The association between meat and mental health among preschoolers is scarce. Investigational results focusing on adolescents and adults are controversial (46, 47). In our study, meat/poultry/fish/eggs were significantly related to preschoolers' mental health. More types of meat per week (≥5 types/week) resulted in better mental health for boys and girls. So does the dairy/soy/nuts. Dairy/soy/nuts, whose benefit for health has been verified by many investigations, were also associated with protective effects on mental health in our study and agreed with a study focused on higher education students in the United States (48). Vegetable oil is rich in unsaturated fatty acids, the effect of which has been a research hotspot in recent years due to its proposed health benefits. We suggest that an adequate vegetable oil intake may be beneficial for boys' mental health, not for girls. The findings might lie in the sex difference in physiological and metabolic characteristics. Overall, further studies on the specific types of food consumption and their relationship with mental health among preschoolers should be further investigated.

Processed meat is well known to be detrimental to physical health, being listed as a Category 1 “carcinogen to humans” according to WHO's International Agency for Research on Cancer. In the present study, we confirmed the harmful effect of the frequency of consuming specific types of processed meat on the mental health of children, which is the first study to explore its association among preschoolers.

The contribution of confectionery to dental health and cardiometabolic diseases like obesity and related outcomes in children has been studied by many studies (49), with disputed effects of these associations. Confectionery in children age 10 has been associated with violence in adults (50). However, no study has investigated its relationship with child mental health. Interestingly, we found that adequate intake of confectionery, not high or low, was associated with protective effects on children's mental health. This may have something to do with the satisfaction that confectionery can bring to children, which longitudinal research should further assess.

The mechanism of food and mental health among children studied possible pathogenic mechanisms and included (51) micro-and macronutrient intake; hormonal homeostasis in the context of glucose metabolism and adiponectin regulation and its impact on systemic and neuroinflammation; individual bioactive molecules exerting antioxidant activities and acting as anti-inflammatory agents, such as omega-3 fatty acids and polyphenols, considered beneficial for the central nervous system *via* modulation of adult neurogenesis, synaptic and neuronal plasticity, and microglia activation; the regulation of the gut-brain axis and its effect on the modulation of systemic inflammation and oxidative stress has been provided.

The present study has several strengths. Apart from its large number of population-based and multinational participants, our study offers the possibility to investigate the parent-reported overall view of their child's diet and adherence with SDQ total and subscale scores concerning mental health. An added strength of the study is the inclusion of a wide range of potentially confounding variables such as parental educational level, nation, residence, number of siblings, physical activity and screen time, BMI status, and other lifestyle behaviors. However, as in all such studies, we may not have adequately accounted for these factors, and residual confounding may explain these findings. Another strength of this study was utilizing the well-validated mental health scales for parents and teachers.

However, our study also has some noted limitations. Firstly, the cross-sectional nature of our study limited the interpretation of the causal relationship between food categories and mental health problems, i.e., whether food categories affect their mental health or whether mental health affects their food choices. Secondly, our study's diet questionnaires did not provide exact data on food intake. The food frequency questionnaire is preferable for extensive studies and collects portion size information as standardized portions. Due to our study design, we had to rely solely on parental reports of diet intake, frequency range, and reporting biases that may limit our results' generalization. And no test-retest precision data for the assessments of food intake was also the limitations of the present study. Thirdly, parental psychopathology, family management, and parental style are likely to impact both diet and mental health in childhood and were not included as confounders but might affect our results. Additionally, the dietary reports may have been influenced by parental under-reporting unhealthy items or over-reporting healthy food items, as parents may not know what their children eat outside the home. However, to control for this potential limitation, the dietary intake frequencies that the parents reported were almost all validated by preschoolers' teachers to improve the quality of our data.

## Conclusion

We have shown a significant association between types of food and mental health issues among Chinese preschoolers, independent of BMI categories, outdoor activity, screen time, and background characteristics. Our findings support the proposition that diverse food categories and adequate frequency of confectionery are associated with better mental health in pre-schoolers. In comparison, processed meat is associated with poorer mental health. Our findings provide new ideas and supportive data for the scheme of children's diet guidelines and improving mental health. Considering the cross-sectional nature of this study, follow-up and intervention studies are needed to investigate the possible relationship between food groups and mental health among pre-schoolers.

## Data availability statement

The raw data supporting the conclusions of this article will be made available by the authors, without undue reservation.

## Ethics statement

The studies involving human participants were reviewed and approved by the Ethics Committee of Zunyi Maternal and Child Health Care Hospital (No. 202110). Written informed consent to participate in this study was provided by the participants' legal guardian/next of kin.

## Author contributions

ZLi, HX, and QJ designed the study and contributed to the interpretation of data. QJ and YL wrote the main manuscript text. HX, JM, QJ, and YL carried out the statistical analysis. QJ, YL, YX, and ZLiu collected the data. ZLi supervised the manuscript. HX revised the manuscript. All authors reviewed the manuscript, read, and approved the final manuscript.

## Funding

This project was founded by the research grant from Guizhou Provincial Health and Health Committee Science and Technology Fund Project (gzwkj2022-201), Science and Technology Plan Project of the Science and Technology Bureau of Honghuagang District, Zunyi City, Guizhou Province ([2021] No. 08), the Natural Science Foundation of Hebei Province (No. H2021201054), Science and Technology Research Project of Higher Education in Hebei Province (No. QN2019130), National Natural Science Foundation of China (82160616), and Guizhou Medical University Ph.D. Start Fund Project (gyfybsky-2021-22).

## Conflict of interest

The authors declare that the research was conducted in the absence of any commercial or financial relationships that could be construed as a potential conflict of interest.

## Publisher's note

All claims expressed in this article are solely those of the authors and do not necessarily represent those of their affiliated organizations, or those of the publisher, the editors and the reviewers. Any product that may be evaluated in this article, or claim that may be made by its manufacturer, is not guaranteed or endorsed by the publisher.
