# Supplementary material for: Association between food intake and mental health problems among preschoolers in China
Source: Front Public Health. 2022 Oct 19;10:1003416. doi: 10.3389/fpubh.2022.1003416 (PMC9627167; doi:10.3389/fpubh.2022.1003416)
Supplement: Supplementary file 1 [file Table_1.DOCX]

**遵义市幼儿身心健康动态发育评估（2021.8）[复制]**

尊敬的家长：
      您好！学前儿童处于心身发育快速时期，也常出现饮食、睡眠、情绪与行为等方面的问题，为促进孩子的心身健康情况，我们特意进行了此次调查。请对孩子的情况比较了解的家长填写。
      问卷题目量较大，请选择一个相对充裕和安静的时间填写。您完整填写这份调查并提交后，会自动生成一份关于儿童睡眠、营养等方面的指导建议，供您参考。
        请注意：本次调查没有设置任何抽奖活动，如果提交后弹出相关广告，敬请忽视！
                           遵义市幼儿身心健康评估小组（遵义医科大学应用心理学教研室&遵义市妇幼保健院儿童保健科）
                                                                                                         2021.8.20

**以下是您孩子的膳食状况，请根据孩子平时的实际情况填写。请注意区别选项是每天还是每周。**

1-A.每天食用谷薯类食物的种类？（谷薯类食物包括大米、小麦、高粱、玉米、小米、薏米、土豆、红薯、山药、红豆、豌豆等） [单选题] *

| ○1-2种 | ○3-4种 | ○5种及以上 |
| --- | --- | --- |

1-B.每周食用谷薯类食物的种类？（谷薯类食物包括大米、小麦、高粱、玉米、小米、薏米、土豆、红薯、山药、红豆、豌豆等） [单选题] *

| ○1-2种 | ○3-4种 | ○5种及以上 |
| --- | --- | --- |

2-A.每天蔬菜和水果一共有几种？ [单选题] *

| ○1-3种 | ○4-6种 | ○7种及以上 |
| --- | --- | --- |

2-B.每周蔬菜和水果一共有几种？ [单选题] *

| ○1-3种 | ○4-6种 | ○7-9种 |
| --- | --- | --- |
| ○≥10种 |  |  |

3-A.每天瘦畜肉、瘦禽肉、鱼虾、蛋类一共几种？ [单选题] *

| ○≤1种 | ○2-4种 | ○≥5种 |
| --- | --- | --- |

3-B.每周瘦畜肉、瘦禽肉、鱼虾、蛋类一共几种？ [单选题] *

| ○≤1种 | ○2-4种 | ○≥5种 |
| --- | --- | --- |

4-A.每天奶类（鲜奶、奶粉、奶酪等）、大豆、坚果（花生、瓜子、核桃、腰果、板栗、）一共几种？ [单选题] *

| ○≤1种 | ○2-4种 | ○≥5种 |
| --- | --- | --- |

4-B.每周奶类（鲜奶、奶粉、奶酪等）、大豆、坚果（花生、瓜子、核桃、腰果、板栗、）一共几种？ [单选题] *

| ○≤1种 | ○2-4种 | ○≥5种 |
| --- | --- | --- |

5-A.每天所有食物一共几种？（包括所有粮食、蔬菜水果、鱼禽肉蛋奶、坚果大豆，不包括油盐及其它调味品） [单选题] *

| ○≤6种 | ○7-11种 | ○12种及以上 |  |  |
| --- | --- | --- | --- | --- |

5-B.每周所有食物一共几种？（包括所有粮食、蔬菜水果、鱼禽肉蛋奶、坚果大豆，不包括油盐及其它调味品） [单选题] *

| ○≤6种 | ○7-11种 | ○12-18种 | ○19-24种 | ○≥25种 |
| --- | --- | --- | --- | --- |

6.吃早餐的次数 [单选题] *

| ○每天都吃 | ○每周4-6次 | ○每周1-3次 | ○每周不足一次 |
| --- | --- | --- | --- |

7.吃三餐的时间规律性 [单选题] *

| ○非常规律 | ○比较规律 | ○一般 |
| --- | --- | --- |
| ○比较不规律 | ○很不规律 |  |

8.通常情况下，家里食用油中植物油（菜油、花生油、核桃油、亚麻油等）占总的食用油（含猪油、牛羊油和植物油）多少比例？   [单选题] *

| ○2/3及以上 | ○1/3至2/3 | ○不足1/3 | ○几乎没有 |
| --- | --- | --- | --- |

9.孩子平时有补充营养素及微生态制剂吗？（可多选） [多选题] *

| □钙 | □铁 | □B组维生素 | □维生素A |
| --- | --- | --- | --- |
| □维生素C | □维生素D | □维生素E | □DHA |
| □硒 | □锌 | □碘 | □叶酸 |
| □益生菌 | □其他 |  |  |

**以下是孩子平时食用饮品的频率（请注意区分选项是每天还是每周）**[矩阵单选题] *

|  | 每天≥3次 | 每天1-2次 | 每周4-6次 | 每周1-3次 | 每周＜1次 |
| --- | --- | --- | --- | --- | --- |
| 1.市售果汁（鲜橙多等） | ○ | ○ | ○ | ○ | ○ |
| 2.碳酸饮料/汽水（可口可乐、雪碧、美年达等） | ○ | ○ | ○ | ○ | ○ |
| 3.咖啡 | ○ | ○ | ○ | ○ | ○ |
| 4.红茶 | ○ | ○ | ○ | ○ | ○ |
| 5.绿茶（花茶） | ○ | ○ | ○ | ○ | ○ |
| 6.其它饮料（津威、红牛、营养快线等） | ○ | ○ | ○ | ○ | ○ |

以下是孩子平时的膳食习惯及膳食频率（请注意区分选项是每天还是每周）[矩阵单选题] *

|  | 每天≥2次 | 每周5-7次 | 每周3-4次 | 每周1-2次 | 每周＜1次 |
| --- | --- | --- | --- | --- | --- |
| 1.菌藻类（蘑菇、木耳、紫菜、海带，等） | ○ | ○ | ○ | ○ | ○ |
| 2.动物内脏（肝脏、心、肚、肠等） | ○ | ○ | ○ | ○ | ○ |
| 3.油炸食品 | ○ | ○ | ○ | ○ | ○ |
| 4.烧烤 | ○ | ○ | ○ | ○ | ○ |
| 5.烟熏食品（腊肉、香肠、熏鱼、熏鸡、熏鸭等） | ○ | ○ | ○ | ○ | ○ |
| 6.泡菜、榨菜、腐乳等腌制品 | ○ | ○ | ○ | ○ | ○ |
| 7.糕点（如绿豆糕/沙琪玛/桃酥等） | ○ | ○ | ○ | ○ | ○ |

您辛苦了！还有最后一小部分，请再接再厉！

为了进行数据的前后对照，邀请您留下您的QQ号或者微信号（若不确定，可都留下）或者电话号码，以便对您的孩子的心身健康发育情况进行动态监测，您的信息会被严格保密。 [填空题] *

_________________________________
